# Effectiveness of Stereotactic Body Radiotherapy for Hepatocellular Carcinoma with Portal Vein and/or Inferior Vena Cava Tumor Thrombosis

**DOI:** 10.1371/journal.pone.0063864

**Published:** 2013-05-30

**Authors:** Mian Xi, Li Zhang, Lei Zhao, Qiao-Qiao Li, Su-Ping Guo, Zi-Zhen Feng, Xiao-Wu Deng, Xiao-Yan Huang, Meng-Zhong Liu

**Affiliations:** State Key Laboratory of Oncology in Southern China, Department of Radiation Oncology, Cancer Center, Sun Yat-sen University, Guangzhou, China; Yonsei University College of Medicine, Korea

## Abstract

**Background:**

To report the feasibility, efficacy, and toxicity of stereotactic body radiotherapy (SBRT) for the treatment of portal vein tumor thrombosis (PVTT) and/or inferior vena cava tumor thrombosis (IVCTT) in patients with advanced hepatocellular carcinoma (HCC).

**Materials and methods:**

Forty-one patients treated with SBRT using volumetric modulated arc therapy (VMAT) for HCC with PVTT/IVCTT between July 2010 and May 2012 were analyzed. Of these, 33 had PVTT and 8 had IVCTT. SBRT was designed to target the tumor thrombosis and deliver a median total dose of 36 Gy (range, 30–48 Gy) in six fractions during two weeks.

**Results:**

The median follow-up was 10.0 months. At the time of analysis, 15 (36.6%) achieved complete response, 16 (39.0%) achieved partial response, 7 (17.1%) patients were stable, and three (7.3%) patients showed progressive disease. No treatment-related Grade 4/5 toxicity was seen within three months after SBRT. One patient had Grade 3 elevation of bilirubin. The one-year overall survival rate was 50.3%, with a median survival of 13.0 months. The only independent predictive factor associated with better survival was response to radiotherapy.

**Conclusions:**

VMAT-based SBRT is a safe and effective treatment option for PVTT/IVCTT in HCC. Prospective randomized controlled trials are warranted to validate the role of SBRT in these patients.

## Introduction

Portal vein tumor thrombosis (PVTT) and inferior vena cava tumor thrombosis (IVCTT) are common complications in patients with advanced hepatocellular carcinoma (HCC). Despite improvement in the survival of HCC, the prognosis of patients with PVTT/IVCTT remains poor, with a median survival of only approximately three months without treatment [1], [2]. PVTT is commonly associated with portal vein hypertension, tumor dissemination, and deterioration of liver function, which then limits the application of surgical resection or transarterial chemoembolization (TACE) on HCC [3], [4].

As no standard treatment modality has been established for HCC with tumor thrombosis, radiotherapy can be considered as an alternative treatment. With the development of radiation techniques such as three-dimensional conformal radiotherapy (3DCRT), intensity-modulated radiotherapy (IMRT), and stereotactic body radiotherapy (SBRT), high-dose radiation can be safely delivered to liver tumors without resulting in serious complications [5]. Several studies have reported the application of 3DCRT in the treatment of HCC with PVTT, which has shown encouraging results in local control and survival [6]–[13]. However, few studies have investigated the efficacy of SBRT for the treatment of PVTT/IVCTT. In addition, the published reports have been limited to small case series, making it difficult to carry out reliable analysis [14], [15].

The purpose of the current study was to report our institutional experience with a relatively large group of patients and to evaluate the feasibility, efficacy, and toxicity of SBRT for PVTT/IVCTT in HCC.

## Materials and Methods

### Ethics statement

This study was approved by our Institutional Review Boards (IRBs) for Cancer Center, Sun Yat-sen University. Written informed consents were obtained from all the patients in accordance with the regulations of IRBs.

### Patient population

We retrospectively reviewed the records of 41 advanced HCC patients with PVTT and/or IVCTT who had received SBRT between July 2010 and May 2012 at our institution. The inclusion criteria were as follows: (1) patients between 20–70 years of age; (2) histopathologically or radiologically diagnosed as having HCC with PVTT/IVCTT, unresectable or medically unsuitable for resection; (3) Child-Pugh class A liver function; (4) more than 800 cc of uninvolved liver; (5) no history of radiotherapy for the liver; and (6) Eastern Cooperative Oncology Group (ECOG) performance status scale ≤2. Patients who had distant metastasis were excluded from this study. PVTT/IVCTT was diagnosed on the basis of a filling defect in the PV/IVC on contrast-enhanced CT or MRI.

Patient and tumor characteristics are listed in Table 1. The median age at diagnosis of PVTT/IVCTT was 54 years (range, 27–70 years). Thirty-four patients (82.9%) had hepatitis B infection, and only one (2.4%) has hepatitis C. Tumor thrombosis involved the first or second order PV branches in 16 patients (39.0%), the main trunk of PV in 17 patients (41.5%), and IVC in 8 patients (19.5%), respectively. For the tumor thrombosis located in both branches and the main trunk of PV (11 patients), we categorized the tumor thrombosis as main trunk.

**Table 1:** Patient and tumor characteristics (n = 41).

Characteristic	No. of patiens	%
Sex		
Male	37	90.2
Female	4	9.8
Age, y		
Median	54	
Range	27–70	
Diagnosis of HCC		
Biopsy	18	43.9
Imaging and AFP	23	56.1
ECOG performance status		
0–1	38	92.7
2	3	7.3
Liver disease		
Hepatitis B	34	82.9
Hepatitis C	1	2.4
No hepatitis	6	14.6
Intrahepatic tumor type		
None	6	14.6
Solitary	12	29.3
Multiple	23	56.1
Abdominal lymph node metastasis		
Yes	11	26.8
No	30	73.2
AFP elevation		
Yes	30	73.2
No	11	26.8
Site of tumor thrombosis		
Portal vein branch	16	39.0
Portal vein trunk	17	41.5
Inferior vena cava	8	19.5
Previous treatment		
TACE	15	36.6
RFA	6	14.6
TACE + RFA	10	24.4
Surgery	10	24.4
Combined with sorafenib		
Yes	14	34.1
No	27	65.9

*Abbreviations:* HCC, hepatocelular carcinoma; AFP, alpha-fetoprotein; ECOG, Eastern Cooperative Oncology Group; TACE, transarterial chemoembolization; RFA, radiofrequency ablation.

In terms of treatment before radiotherapy, 25 patients had received median 2 cycles of TACE (range, 1–6 cycles) with or without radiofrequency ablation (RFA). Ten patients had been treated with resection for intrahepatic tumors before the occurrence of tumor thrombosis. For the other 6 patients, RFA was performed as the initial treatment before SBRT. Regarding the treatment of TACE, hepatic artery infusion chemotherapy was performed using carboplatin 300 mg; next, chemolipiodolization was performed using epirubicin 50 mg and mitomycin 8 mg mixed with 5 mL of lipidol. Of the 41 patients, 14 (34.1%) received oral sorafenib (400 mg bid) at least one month before radiation. The targeted therapy was continued during and after radiotherapy until disease progression.

### Radiation treatment

Patients were immobilized with vacuum bags in the supine position with the arms raised above the head during simulation. Contrast-enhanced four-dimensional computed tomography (4DCT) scans were acquired at 2.5-mm slice thickness on a 16-slice positron emission tomography PET/CT (GE Medical Systems, Waukesha, WI) during uncoached, quiet breathing [16].

The gross tumor volume (GTV) represented the tumor thrombosis visualized on the CT images, and/or near primary liver lesions and/or positive abdominal lymph node (LN) metastasis. Internal target volume (ITV) was defined as the combined volume of GTVs in the multiple 4DCT phases. An isotropic margin of 0.6 cm was added to ITV to account for interfractional motion variability and daily setup errors in order to generate a planning target volume (PTV). Organs at risk (OARs) included the liver, kidneys, stomach, small intestine, and spinal cord. Normal liver volume was defined as the total liver volume minus the GTV.

Volumetric modulated arc therapy (VMAT) was planned for an Elekta Synergy accelerator (MLCi2, 80 leaves; width, 1 cm) with 8 MV photons. VMAT planning was performed using Monaco TPS (CMS, Elekta, version 3.0) and consisted of a single 360° arc, which uses simultaneous variation of gantry rotational speed, MLC leaf positions, and dose rate to optimize the dose distribution, as previously described [17].

The median prescription dose was 36 Gy (range, 30–48 Gy) to PTV in six fractions administered over two weeks. Planning objectives for the PTVs aimed to limit the minimal and maximal doses to 90% and 110% of the prescribed dose. Dose-volume planning objectives for the OARs were defined as follows: normal liver, mean dose ≤18 Gy; stomach, maximal dose ≤35 Gy; small intestine, maximal dose ≤30 Gy; bilateral kidney, mean dose ≤18 Gy; and spinal cord, maximal dose <27 Gy. For the stomach and small intestine, the maximal dose was expressed as D_0.5cc_.

Regarding the quality of VMAT delivery and the agreement between the dose calculations and treatment, the VMAT plans were verified dosimetrically using a Delta 4 phantom (ScandiDos, Uppsala, Sweden) before treatment, as described by Bedford et al. [18]. The gamma evaluation criterion was ±3% of 2 Gy and the distance to agreement was 3 mm, as commonly used in the clinic. In each fraction of SBRT delivery, online couch adjustment using kilovoltage cone-beam CT scans was performed for isocenter verification.

### Evaluation

Patients were assessed for toxicities on a weekly basis during SBRT and once every three months thereafter. Treatment-associated acute and late toxicities were scored according to the Common Terminology Criteria for Adverse Events (CTCAE; version 3.0). Tumor response was assessed using the modified Response Evaluation Criteria in Solid Tumors (mRECIST) criteria [19]. The response of PVTT/IVCTT to SBRT was evaluated by contrast-enhanced spiral CT scans performed three months after completion of radiotherapy. Biochemical response was assessed in patients with elevated alpha-fetoprotein (AFP) level before radiotherapy and defined by either a ≥50% reduction or normalization of the AFP level within three months after SBRT.

### Follow-up and statistical analysis

The cutoff date for the last follow-up was November 30, 2012, for the censored data analysis. The overall survival (OS) was calculated from the start of radiotherapy to the date of either death or the last follow-up visit. The Kaplan-Meier method was used to analyze the OS, the log-rank test was used to examine group differences, and a Cox regression model was used for multivariate analysis. All statistical analyses were performed using the SPSS software package (version 13.0; SPSS Inc., Chicago). A *P* value of <0.05 was considered statistically significant.

## Results

### Technical and dosimetric findings

The mean GTV and PTV volumes were 65.4±47.9 and 201.8±115.0 cc, respectively. The mean normal liver volume was 1252.6±257.6 cc. The planning objectives were reached in all VMAT plans and all cases completed the planned radiotherapy. The mean doses administered to the normal liver and bilateral kidney were 12.7±2.6 and 3.9±1.8 Gy, respectively. The maximal dose to the serial OARs were as follows: 25.8±6.7 Gy for stomach, 17.5±11.3 Gy for small intestine, and 16.6±5.2 Gy for spinal cord. The number of monitor units (MU) per fraction for VMAT plans was 1270±189, and the average effective treatment time was 3.9±0.6 min. The average gamma evaluation passing rate of VMAT delivery was 98.6±1.2%.

### Tumor response

**Figure 1:**
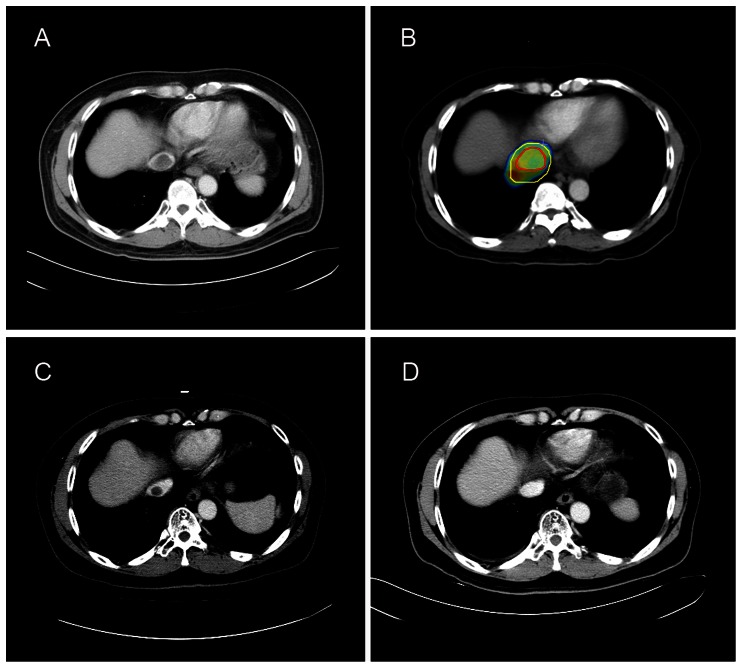
A HCC patient with IVCTT demonstrating a CR after radiotherapy. (A) Pre-treatment image. (B) Dose distribution in the axial view. The red and yellow contours represent the GTV and PTV, respectively. (C) One month after SBRT. (D) Three months after SBRT.

Evaluating response according to mRECIST criteria, complete response (CR) was achieved in 15 (36.6%); partial response (PR), in 16 (39.0%); stable disease (SD), in 7 (17.1%); and progressive disease (PD), in 3 patients (7.3%), yielding an objective response rate (CR + PR) of 75.6%. Typical presentations before and after SBRT are illustrated in Figure 1. Of the 30 patients with elevated AFP levels before radiotherapy, 23 patients (76.7%) exhibited ≥50% reduction in the AFP levels within three months after SBRT.

### Follow-up and survival

Three months after completion of SBRT, 15 patients received additional 1–4 cycles of TACE and 5 patients received further RFA because of the restoration of PV patency or multiple intrahepatic lesions. Three patients underwent surgical resection 3.2 months, 4.1months, and 5.9 months after radiotherapy, respectively. Two of them died from surgery-related complications and the other one patient is still alive without evidence of disease.

The median follow-up period was 10.0 months (range, 3.6–25.3 months). During follow-up, PD was noted in 28 patients (14 with intrahepatic progression, 2 with distant metastasis, and 12 patients with both). The majority (12/14) of intrahepatic progression occurred outside the treated volume. Lungs were the most frequent site of distant metastasis followed by the brain and adrenal gland.

**Figure 2:**
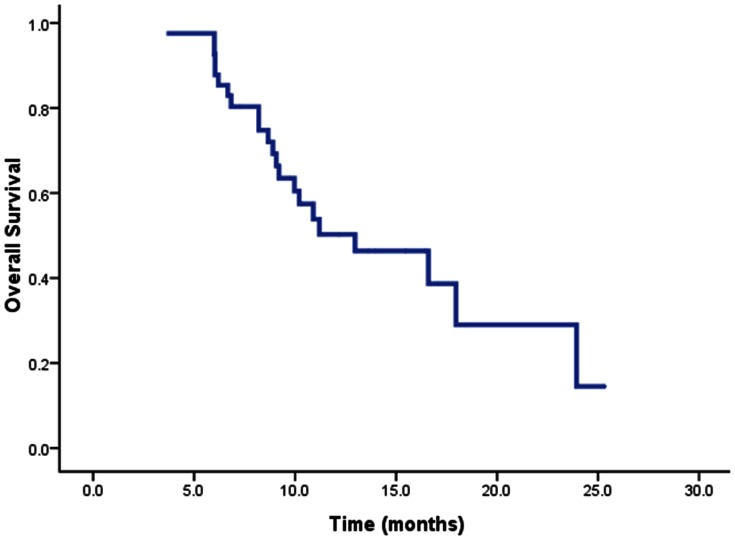
Overall survival curve for the whole group of 41 HCC patients with PVTT/IVCTT.

**Figure 3:**
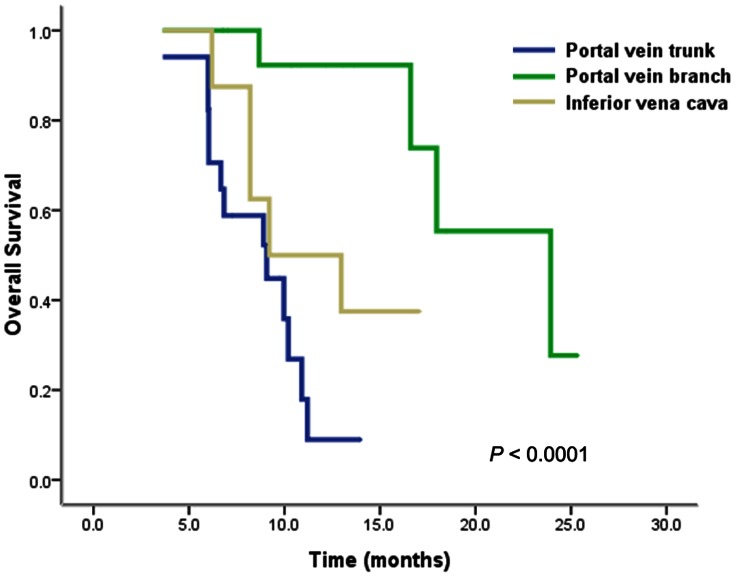
Overall survival curves according to the site of tumor thrombosis. Patients with PVTT in branches had longer survival than those with PVTT in main trunk or IVCTT (*P*<0.0001).

Nineteen patients (46.3%) survived at the time of the current analysis, including eight without disease and 11 with disease progression. The median survival time for the whole group was 13.0 months (95% CI, 7.1–18.8 months), with a 1-year OS rate of 50.3% (Fig. 2). The median survival of PVTT in branches, PVTT in main trunk, and IVCTT were 23.9 months (95% CI, 14.6–33.3 months), 9.1 months (95% CI, 5.1–13.0 months), and 9.2 months (95% CI, 2.6–15.8 months), respectively (Fig. 3).

### Predictors of survival

**Figure 4:**
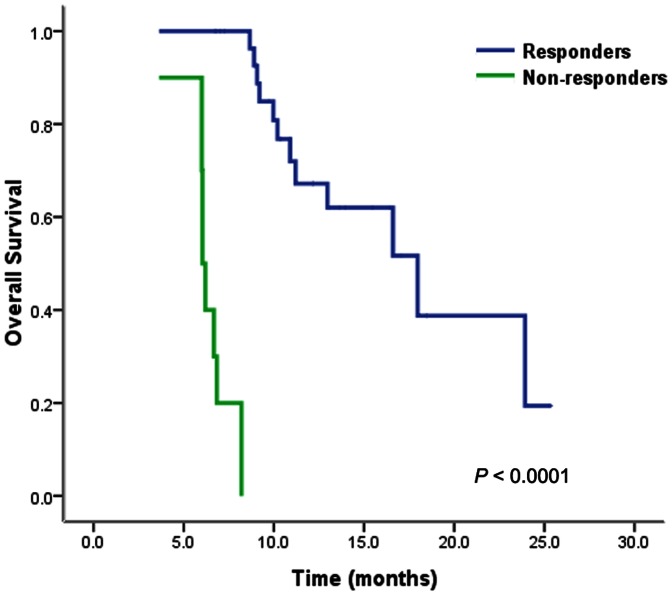
Overall survival curves according to the response to radiotherapy of PVTT/IVCTT. Responders had significantly better survival than non-responders (*P*<0.0001).

Univariate analysis revealed that limited intrahepatic tumor (≤1), well-controlled intrahepatic lesion, absence of LN metastasis, PVTT in branches, higher radiation dose (≥36 Gy), and response to radiotherapy were favorable prognostic indicators of survival (Table 2). In the multivariate analysis, response to radiotherapy was the only independent predictive factor associated with better OS (*P* = 0.043). The median OS of responders and non-responders were 18.0 months (95% CI, 11.8–24.2 months) and 6.0 months (95% CI, 5.8–6.2 months), respectively (*P*<0.0001; Fig. 4).

**Table 2:** Analysis of prognostic factors for survival.

Factor	N	Median survival (month; 95% CI)	*P* values	
			Univariate	Multivariate
Sex				
Male	37	13.0 (6.5–19.4)	0.756	
Female	4	10.2 (8.1–12.3)		
Age, y				
<54	17	16.6 (6.2–27.1)	0.847	
≥54	24	13.0 (8.3–17.7)		
AFP				
<400	21	10.9 (7.1–18.8)	0.956	
≥400	20	13.0 (5.2–20.8)		
Intrahepatic tumor type				
None/solitary	18	23.9 (17.0–30.8)	<0.0001	0.610
Multiple	23	8.9 (7.7–10.1)		
Intrahepatic lesion control				
Well-controlled	8	17.6 (15.9–19.2)	0.013	0.532
Uncontrolled	33	10.0 (7.9–12.1)		
Abdominal LNM				
Yes	11	8.2 (6.0–10.4)	<0.0001	0.295
No	30	18.0 (11.8–24.1)		
Site of tumor thrombosis				
Portal vein branch	16	23.9 (14.6–33.3)	<0.0001	0.879
Portal vein trunk	17	9.1 (5.1–13.0)		
Inferior vena cava	8	9.2 (2.6–15.8)		
Combined with sorafenib				
Yes	14	16.6 (10.1–23.1)	0.755	
No	27	10.9 (5.7–16.1)		
Radiation dose				
<36 Gy	11	8.2 (5.8–10.6)	0.001	0.964
≥36 Gy	30	16.6 (11.2–22.0)		
Response to RT				
CR + PR	31	18.0 (11.8–24.2)	<0.0001	0.043
SD + PD	10	6.0 (5.8–6.2)		
Additional treatment after RT				
Yes	23	10.9 (5.4–16.4)	0.551	
No	18	11.2 (6.0–17.1)		

*Abbreviations:* AFP, alpha-fetoprotein; LNM, lymph node metastasis; RT, radiotherapy; CR, complete response; PR, partial response; SD, stable disease; PD, progressive disease.

### Toxicity

As shown in Table 3, no treatment-related Grade 4 or 5 acute toxicity was seen within three months after SBRT. Only one patient (2.4%) showed Grade 3 elevation of bilirubin. Grade 1 nausea/vomiting was the most common toxicity encountered during SBRT. Three patients (7.3%) developed late toxicity: one patient exhibited Grade 1 liver enzyme elevation, and two patients experienced Grade 2 decrease in their platelet counts.

**Table 3:** Acute toxicity.

CTC toxicity	No. of patiens	%
Nausea/vomiting, grade		
0	5	12.2
1	30	73.2
2	6	14.6
3–5	0	0
Liver enzymes, grade		
0	23	56.1
1	16	39.0
2	2	4.9
3–5	0	0
Bilirubin, grade		
0	31	75.6
1	8	19.5
2	1	2.4
3	1	2.4
4–5	0	0
Anemia		
0	33	80.5
1	8	19.5
2–5	0	0
Leukocytes		
0	25	61.0
1	12	29.3
2	4	9.8
3–5	0	0
Platelets		
0	20	48.8
1	12	29.3
2	9	22.0
3–5	0	0

*Abbreviations:* CTC, common toxicity criteria.

## Discussion

SBRT has shown encouraging rates of local control and low toxicity for HCC and hepatic metastasis [20], [21]. However, very few studies have investigated SBRT for PVTT/IVCTT in HCC patients. The efficacy and toxicity of SBRT for these patients have not been well documented previously. This study demonstrated that SBRT is an effective treatment modality with a low incidence of severe side effects for advanced HCC with PVTT/IVCTT.

With advances in radiotherapy techniques, 3DCRT has proved its efficacy in HCC with vascular invasion. The overall treatment response rate from the series of 3DCRT was 25.2–62.3% [6]–[13]. The 1-year overall survival (OS) rate was 25.0–57.6%, with a median survival time of 3.8–13.9 months [6]–[13]. However, most of the prior published reports of 3DCRT have relatively small number of patients. In one large series from Korea consisting of 412 HCC with PVTT, the patients were treated with 40 (range, 21–60) Gy in daily fractions of 2–5 Gy [8]. The response rate of PVTT was 39.6%. The median survival was 10.6 months, and the 1-year OS rate was 42.5%. Huang et al. [12] reported the worst response rate of 25.2% for PVTT treated with 3DCRT or IMRT. For the 326 patients, the median survival time was only 3.8 months, with the one-year OS rate of 16.7%. The best survival outcome was reported by Rim et al. [10], who reported that the CR and PR rates for 45 HCC patients with PVTT were 6.7% and 55.6%, respectively. As for the limited studies reporting the efficacy of SBRT for PVTT, the median OS was only 6–8 months for fewer than 10 patients [14], [15]. In the present study, the objective response rate was 75.6%, with a median survival of 13.0 months, which was at the higher end of the wide range reported previously. In addition, our results are similar to other reports stating that response to radiotherapy was associated with better survival [10]–[13]. The median OS of patients with objective response (18.0 months) in our series was better than that in most previous studies (10.7–19.9 months) [10]–[13]. The difference in the response rates and survival between our study and others may be caused by different radiation techniques, variation in dose schedules, different response evaluation criteria, as well as heterogeneous eligibility criteria for radiotherapy.

The majority of previous reports of 3DCRT for HCC used 1.8–3.0 Gy daily to achieve total doses of 30–60 Gy [6]. However, at present, the optimal dose fractionation schedule for SBRT is still unclear. Tse et al. [20] reported good outcomes for unresectable HCC and intrahepatic cholangiocarcinoma treated with SBRT in six fractions administered during two weeks. Therefore, we adopted this dose schedule for the treatment of PVTT/IVCTT in our study. Our study showed that six-fraction SBRT was well tolerated by HCC patients with PVTT/IVCTT, without the occurrence of related serious toxicities. The incidence of Grade 3 acute toxicity in our cohort (2.4%) was lower than that reported in previous 3DCRT series [7], [8], [13].

Although SBRT is a promising therapeutic strategy for HCC with tumor thrombosis, survival after radiotherapy remains limited due to the high frequency of intra- and extra-hepatic recurrences. In theory, the combination of radiotherapy and systemic therapy may provide clinical benefits for patients with PVTT/IVCTT. Sorafenib, an orally active multi-kinase inhibitor, is the only systemic agent that has demonstrated an improved OS in HCC patients [22], [23]. In the current study, 14 patients (34.1%) received combining therapy of sorafenib and SBRT. The group that received this treatment showed a favorable trend in median survival (16.6 *vs.* 10.9 months); however, it was not statistically significant (*P* = 0.755). The limited number of patients who received sorafenib in our study may affect the analysis of its therapeutic efficacy; thus, a prospective study addressing this combination is warranted.

Robotic non-isocentric dedicated linac systems are widely used for SBRT in the thoracic and abdominal regions. However, current clinical experience with VMAT-based SBRT is still scarce. A unique feature of this study is the combination of SBRT with VMAT, which is a new development in multileaf collimator-based linac radiation delivery. VMAT is a novel extension of the standard IMRT technique, allowing dose delivery with simultaneously varying gantry speed, MLC shape, and dose rate. The major advantages of VMAT over classic IMRT are the lower number of MUs and the higher delivery efficiency, with a reduction of 35–61% in treatment time [24]. The average treatment time was 3.9±0.6 min for VMAT in our study, which is similar to that reported by Scorsetti et al. [25]. Taking into account the plan quality, treatment efficiency, and delivery accuracy, VMAT-based SBRT can be considered clinically feasible for the treatment of HCC with PVTT/IVCTT.

In conclusion, our study suggests that VMAT-based SBRT is a very safe and effective treatment option for PVTT/IVCTT in HCC patients. Prospective randomized controlled trials are required to further confirm the role of SBRT in these patients.
